# Long-term survival without recurrence after surgery for gastric yolk sac tumor-like carcinoma: a case report

**DOI:** 10.1186/s40792-021-01199-3

**Published:** 2021-05-06

**Authors:** Hibiki Umeda, Satoru Kikuchi, Shinji Kuroda, Shuya Yano, Takehiro Tanaka, Kazuhiro Noma, Masahiko Nishizaki, Shunsuke Kagawa, Yuzo Umeda, Toshiyoshi Fujiwara

**Affiliations:** 1 Department of Gastroenterological Surgery, Okayama University Graduate School of Medicine, Dentistry, and Pharmaceutical Sciences, 2-5-1 Shikata-cho, Kita-ku, Okayama, 700-8558 Japan; 2 Department of Pathology, Okayama University Graduate School of Medicine, Dentistry and Pharmaceutical Sciences, 2-5-1 Shikata-cho Kita-ku, Okayama, 700-8558 Japan

**Keywords:** Gastric yolk sac Tumor-like carcinoma, Adenocarcinoma, Alpha-fetoprotein

## Abstract

**Background:**

Gastric yolk sac tumor (YST)-like carcinoma is extremely rare, and its prognosis is poor, because most patients have widespread metastases at the time of diagnosis. We report a case of gastric YST-like carcinoma with an adenocarcinoma component without metastases in which curative resection was performed.

**Case presentation:**

A 77-year-old man complaining of melena and dizziness due to anemia was diagnosed with poorly differentiated adenocarcinoma in the gastric cardia, with a benign ulcer in the gastric body. He underwent total gastrectomy with D2 lymph node dissection for the tumor. Histological examination of the resected specimens revealed a mixture of reticular and glandular neoplastic components morphologically. In the reticular area, an endodermal sinus pattern and some Schiller–Duval bodies were confirmed. Gastric YST-like carcinoma with adenocarcinoma components, T2N0M0 Stage IB, was diagnosed. Immunohistochemical analysis showed that the YST was positive for carcinoembryonic antigen (CEA), alpha-fetoprotein (AFP) and p53. In contrast, the adenocarcinoma was positive for p53 and negative for CEA and AFP. The patient remained healthy as of 7 years postoperatively, with no recurrence.

**Conclusions:**

Routine medical examinations or endoscopic examinations for accidental symptom may be helpful for early diagnosis and good prognosis for gastric YST-like carcinoma, although the prognosis is generally poor.

## Background

Yolk sac tumors (YSTs) are germ cell tumors, which usually arise in the gonads, but can also occur in extragonadal regions such as the sacrococcygeal region, the lungs, the anterior mediastinum, the retroperitoneum, and the brain. These tumors have also been reported to arise as a component in carcinomas with heterogeneous differentiation in the lungs, stomach, large intestine, gallbladder, pancreas, urinary bladder, and so on [1]. Gastric YST-like carcinoma is extremely rare, and only 19 cases have been reported [1–19]. Generally, gastric YST-like carcinoma is a highly aggressive tumor with a poor prognosis, because most patients display lymph node or distant metastases at the time of diagnosis.

Here, we present a case of gastric YST with adenocarcinoma components that was curatively resected, obtaining long-term survival without recurrence for the patient.

## Case presentation

**Fig. 1:**
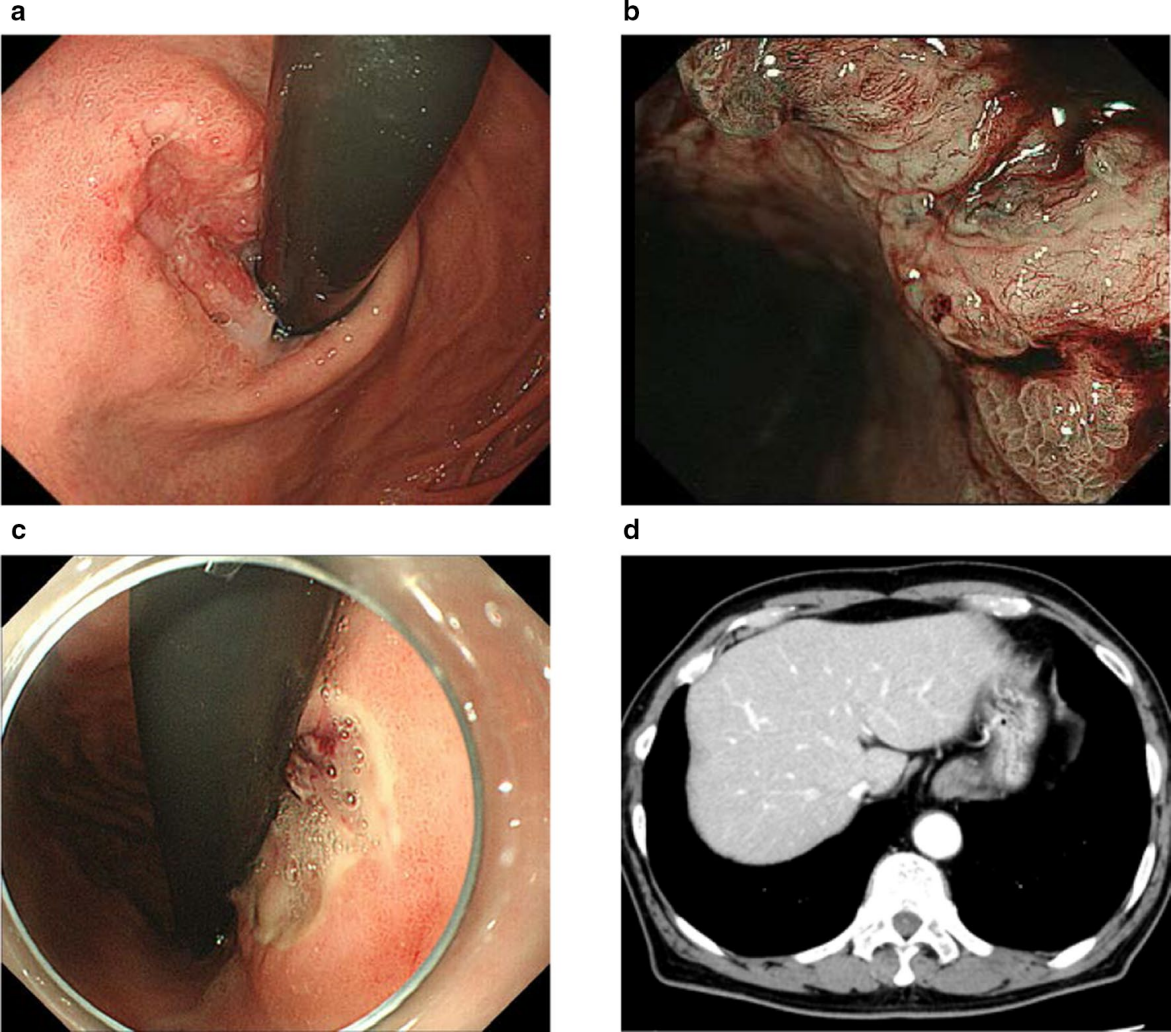
**a** Esophagogastroduodenoscopy shows an ulcerated tumor at the gastric cardia. **b** Narrow-band imaging of tumor shows irregular microvascular and microsurface patterns with wavy micro-vessels. **c** Esophagogastroduodenoscopy also shows an ulcer with exposed vessel at the mid-body of the stomach. **d** Computed tomography of the abdomen and pelvis shows no thickening of the stomach wall and no lymph node enlargement

**Fig. 2:**
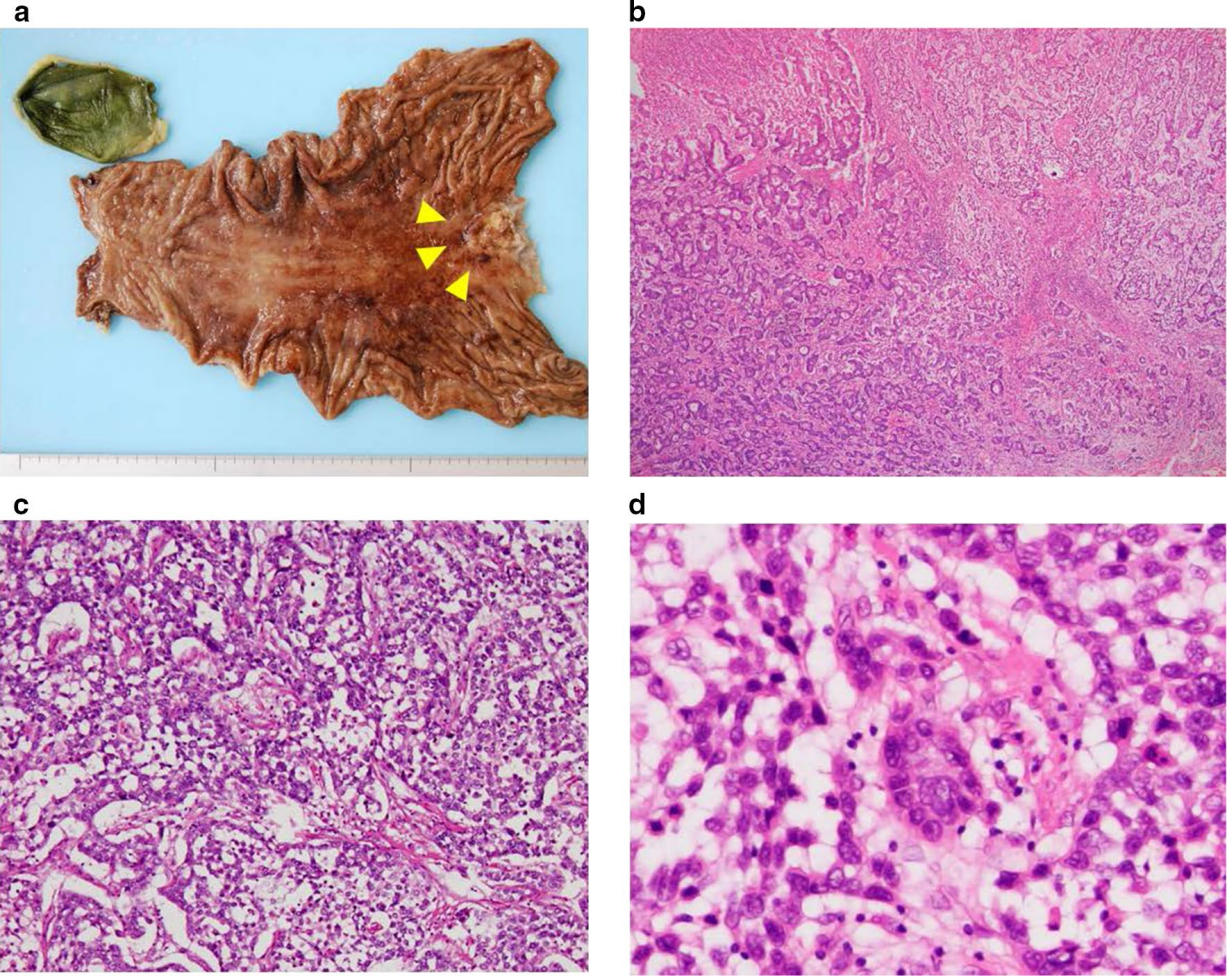
**a** Resected specimens show Borrman type 3 tumor in the cardia (arrowheads). **b**–**d** Histological examination reveals an adenocarcinoma component and a yolk sac tumor (YST) component, with a reticular pattern (**c**) and Schiller–Duval bodies (**d**) are observed in YST component (hematoxylin and eosin staining)

**Fig. 3:**
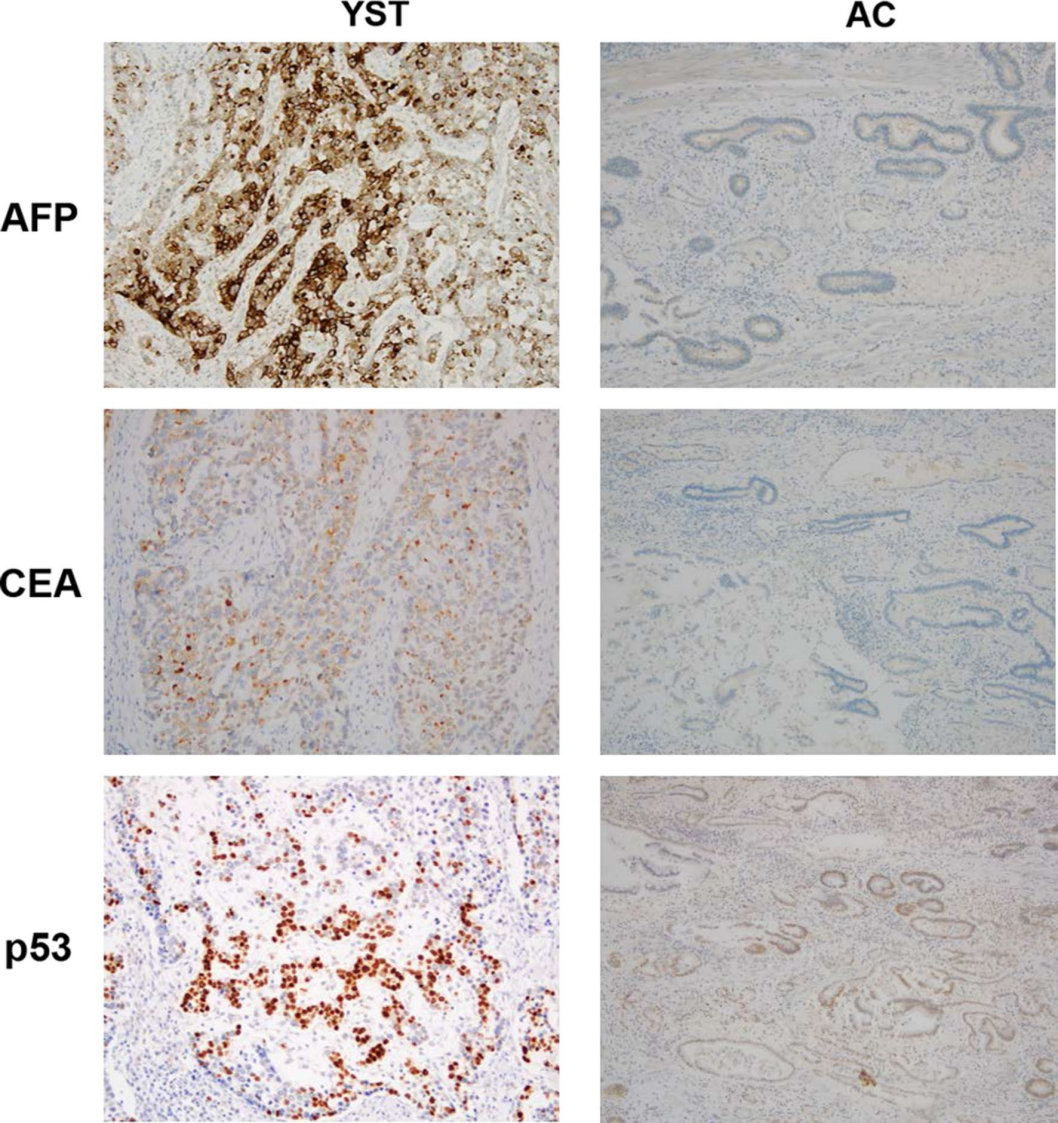
Immunohistochemical staining of the tumor shows the yolk sac tumor (YST) component is positive for alpha-fetoprotein (AFP), carcinoembryonic antigen (CEA) and p53. The adenocarcinoma (AC) component is positive for p53, but negative for AFP and CEA

A 77-year-old man visited a previous hospital complaining of melena and dizziness due to anemia. Endoscopic examination showed an ulcerated tumor at the gastric cardia, clinically suspected to represent gastric cancer (Fig. 1a, b), and an ulcer at the mid-body of the stomach causing active bleeding (Fig. 1c). Biopsy was performed, and histological features of the specimen from the cardia were interpreted as a poorly differentiated adenocarcinoma, while the specimen from the mid-body was a non-neoplastic lesion. The patient was referred to our hospital. Computed tomography (CT) of the abdomen and pelvis did not show any thickening of the stomach wall indicating the tumor, and no lymph node enlargement was found (Fig. 1d). Serum levels of carcinoembryonic antigen (CEA) and carbohydrate antigen 19-9 (CA19-9) were normal. Blood analysis for serum alpha-fetoprotein (AFP) was not performed preoperatively. The patient underwent total gastrectomy with D2 lymph node dissection followed by Roux-en-Y esophagojejunostomy and cholecystectomy (Fig. 2a). Histopathological examination of the resected specimens revealed the presence of two microscopic patterns: an adenocarcinoma component; and a YST component (Fig. 2b). The tumor mostly comprised various areas of differentiated tubular adenocarcinoma. However, a YST component was seen as a lace-like (reticular) network lined by cuboidal to flattened malignant cells and papillary structures (Fig. 2c). Schiller–Duval bodies were also sporadically present (Fig. 2d). The tumor had invaded the muscularis propria, but no lymph nodes metastasis was confirmed, and T2N0M0 Stage IB was diagnosed according to the Japanese classification of gastric carcinoma [20]. Immunohistochemical analysis showed that most of the YST component was positive for AFP, CEA and p53. The adenocarcinoma component was positive for p53, but negative for AFP and CEA (Fig. 3). After the surgery, serum AFP level remained normal. The patient remains healthy and without tumor recurrence as of 7 years postoperatively.

## Discussion

Gastric germ cell tumors are extremely rare; choriocarcinoma arising in the stomach has been reported to comprise less than 0.1% of all gastric cancers and YST-like carcinoma is much rarer than choriocarcinoma [1]. The origin of YST arising in the stomach remains unclear. The main hypothesis is that YST derives from migrating germ cells during embryogenesis, similar to other extragonadal germ cell tumors [6]. This hypothesis is based on the fact that some pure YST-like carcinomas in the stomach have been reported [4, 9, 11–13, 18, 19]. However, several reports have suggested that adenocarcinoma components of the tumor heterogeneously transform to YST through aberrant differentiation, because the majority of gastric YST-like carcinomas are accompanied by an adenocarcinoma component, as observed in the current case [1, 2, 12]. Moreover, Puglisi et al. [6] confirmed identical p53 mutations in both adenocarcinoma and YST in the stomach using molecular analysis, which may support the hypothesis that adenocarcinoma components heterogeneously transform to YST. We did not perform a brain CT and an ultrasound examination of testes to exclude other primary lesions of germ cell tumor. We diagnosed the present case as a primary gastric YST-like carcinoma, because this tumor accompanied with adenocarcinoma component.

Histologically, YST exhibits the feature of an endodermal sinus tumor, including reticular, microcystic, polyvesicular, papillary, solid, or tubercular patterns [19]. The most characteristic finding is Schiller–Duval bodies, as glomerulus-like structures that were also detected in the current case. YST cells often contain hyaline droplets that stain positive for Periodic acid-Schiff. Serum AFP levels generally increase, and the cells stain positively for AFP immunohistochemically, because the YST cells produce AFP. AFP-producing gastric carcinomas are rare, reportedly representing about 1.2% of all gastric carcinomas [5]. Hepatoid adenocarcinoma is the most common AFP-producing gastric carcinoma. Hepatoid adenocarcinoma shows several pathological overlaps with YST, as both glandular and hepatocellular differentiation with AFP production. Schiller–Duval bodies are a salient component distinguishing YST from hepatoid adenocarcinoma.

Twenty cases of gastric YST-like carcinoma, including the current case, have been reported in the medical literature and are summarized in Table 1. This pathology usually affects middle-aged and elderly men and 13 cases (65%) have also been reported to show adenocarcinoma components. It is difficult to expect gastric YST-like carcinoma endoscopically, because there are no characteristic findings in gastric YST-like carcinoma. In the current case, irregular microvascular and microsurface patterns with wavy micro-vessels were observed in narrow-band imaging of endoscopic examination (Fig. 1b), which were characteristic findings of poorly differentiated adenocarcinoma of stomach. YST components were immunohistochemically positive for AFP in all cases, but the adenocarcinoma component was generally negative, as confirmed by the current case. Serum AFP levels were increased in the most cases. Serum AFP level is useful in the diagnosis and surveillance of gastric YST-like carcinoma, but was only measured after surgery in the present case, because we did not measure routinely serum AFP for gastric cancer before surgery, and did not elevate at any point. The prognosis of patients with gastric YST-like carcinoma is generally poor, because most patients have widespread metastases at the time of diagnosis, and most patients die within 1 year after diagnosis. We did not perform adjuvant chemotherapy for the present case according to Japanese gastric cancer treatment guidelines [21]. Although adjuvant chemotherapies including cisplatin, vinblastine, bleomycin, and etoposide according to germ cell tumor treatment were performed for advanced gastric YST-like carcinoma, they have not been shown improve long-term survival [5–7, 13]. The chemotherapy regimens for common type gastric cancer consists of adenocarcinoma might be recommended if it was hypothesized that adenocarcinoma components heterogeneously transform to YST. In the current case, gastric YST-like carcinoma could be detected without metastasis due to occasional bleeding from a gastric benign ulcer concurrent with gastric YST-like carcinoma although the patient had not undergone endoscopic examination before YST-like carcinoma was detected.

**Table 1:** Summary of gastric yolk sac tumor-like carcinomas in the literature

Authors (year)	Age/Sex	Histology	Metastasis	Therapy	Prognosis
Garcia (1985)	65/M	YST, CC, AC	Liver	None	Autopsy case
Motoyama (1985)	72/F	YST, AC	LN	S	Alive (3 years)
Zamecnik (1993)	88/M	YST	LN, Peritoneum	S	Died (4 weeks)
Suzuki (1999)	56/M	YST, AC	LN	S, CT	Died (6 months)
Puglisi (1999)	61/M	YST, AC	Peritoneum	Palliative S	Died (1 months)
Wang (2000)	36/M	YST, AC	LN	CT	Died (6 months)
Napaki (2004)	38/F	YST, AC	Liver	CT, S	Alive (32 months)
Kanai (2005)	87/M	YST	None	S	Died (7 months)
Singh (2007)	67/M	YST, AC	Liver, LN	S, CT	Died (2 months)
Tahara (2008)	74/M	YST	Liver, Lung, LN	None	Died (6 days)
Kim (2009)	61/M	YST	None	S	Alive (3 months)
Magni (2010)	62/M	YST	LN	S, CT	Died (1 year)
(2011)	74/M	YST, CC, AC	Liver, LN	S, RFA, CT	Alive (8 months)
Bihari (2013)	50/M	YST, AC	Liver	None	NM
Yalaza (2017)	68/F	YST, AC	LN	S, CT	Died (8 months)
Lakshmanan (2017)	75/M	YST, AC, HC	None	S	Alive (30 months)
Qureshi (2018)	52/M	YST, AC	LN	S, CT	Alive (16 months)
Mandelia (2018)	3/M	YST	Liver, Peritoneum	CT, S	Alive (5 months)
Ibrahim (2019)	86/F	YST	None	S	NM
Present case	77/M	YST, AC	None	S	Alive (7 years)

*YST* yolk sac tumor, *AC* adenocarcinoma, *CC* choriocarcinoma, *HC* hepatocellular carcinoma, *LN* lymph node, *S* surgery, *CT* chemotherapy, *NM* not mentioned

## Conclusions

A routine or endoscopic examination for accidental symptom may be helpful for achieving early diagnosis and good prognosis for gastric YST-like carcinoma, even though the prognosis is generally poor.

## Data Availability

The datasets supporting this article are included in this paper.

## Abbreviations

YST: Yolk sac tumor
CEA: Carcinoembryonic antigen
AFP: Alpha-fetoprotein
CT: Computed tomography
